# Range Expansion of Tick Disease Vectors in North America: Implications for Spread of Tick-Borne Disease

**DOI:** 10.3390/ijerph15030478

**Published:** 2018-03-09

**Authors:** Daniel E. Sonenshine

**Affiliations:** 1 Laboratory for Malaria and Vector Research, National Institute of Allergy and Infectious Diseases, National Institutes of Health, Rockville, MD 20852, USA; dsonensh@odu.edu or Daniel.Sonenshine@nih.gov; Tel.: +1-757-404-4331; 2 Department of Biological Sciences, Old Dominion University, Norfolk, VA 23529, USA

**Keywords:** climate change, habitats, hosts, *Dermacentor variabilis*, *Amblyomma americanum*, *Amblyomma maculatum*, *Ixodes scapularis*, abiotic factors, biotic factors

## Abstract

Ticks are the major vectors of most disease-causing agents to humans, companion animals and wildlife. Moreover, ticks transmit a greater variety of pathogenic agents than any other blood-feeding arthropod. Ticks have been expanding their geographic ranges in recent decades largely due to climate change. Furthermore, tick populations in many areas of their past and even newly established localities have increased in abundance. These dynamic changes present new and increasing severe public health threats to humans, livestock and companion animals in areas where they were previously unknown or were considered to be of minor importance. Here in this review, the geographic status of four representative tick species are discussed in relation to these public health concerns, namely, the American dog tick, *Dermacentor variabilis*, the lone star tick, *Amblyomma americanum*, the Gulf Coast Tick, *Amblyomma maculatum* and the black-legged tick, *Ixodes scapularis*. Both biotic and abiotic factors that may influence future range expansion and successful colony formation in new habitats are discussed.

## 1. Introduction

Ticks are notorious as vectors of disease-causing agents to humans, companion animals and wildlife. Ticks are responsible for nearly 95% of the vector-borne diseases reported annually in the United States [1]. Among the almost 900 species of ticks [2,3] dispersed globally throughout the world, only about 25 or so are of major medical and veterinary importance. Ticks transmit a greater variety of pathogenic microorganisms than any other hematophagous arthropod, e.g., the agents of Lyme disease, Rocky Mountain spotted fever, human granulocytic anaplasmosis, human monocytic anaplasmosis, tick-borne encephalitis, babesiosis, theileriosis, ehrlichiosis and many others. In addition, tick bites can cause substantial blood loss, severe toxic reactions and even death due to tick paralysis [4]. 

Most of the vector ticks have a well-defined geographic range wherein they are constrained by their adaptations to local abiotic environmental factors, e.g., relative humidity, temperature variations, micro environmental factors such as soil moisture and soil permeability and biotic factors such as dense vegetation, humid leaf litter, and forests providing dense shade. Many species also are constrained by host–parasite associations, especially host-specificity relationships that have evolved over thousands or even millions of years [5]. Ticks spend most of their life cycle off their hosts, limited only by their ability to survive many months and even years without feeding because of their extremely low resting metabolic rates [6] and diapause. Representative examples of species with limited geographic ranges include (1) the American dog tick, *Dermacentor variabilis*, found in brushy and forested habitats throughout most of the central and eastern United States from Florida to several areas of southern Canada; (2) the Rocky Mountain wood tick, *Dermacentor andersoni*, found in the northwestern United States and the Canadian Rocky Mountains; (3) the lone star tick, *Amblyomma americanum*, now present in most regions of the eastern and midwestern U.S.; (4) the Gulf Coast tick, *Amblyomma maculatum*, now present in the Gulf Coast states, midwestern and south Atlantic states of the U.S. In contrast, examples of tick species with very broad (and expanding) geographic and host ranges are the black-legged tick *Ixodes scapularis* in North America, the closely related sheep tick, *I. ricinus*, found mostly throughout most of Europe, United Kingdom and localities in North Africa, and the taiga tick, *I. persulcatus*, found in eastern Europe and northern Asia. 

This review will consider four tick species in North America, *D. variabilis*, *A. americanum*, *A. maculatum* and *I. scapularis*, for an in-depth examination of their geographic range expansion and some of the factors influencing its progress. 

## 2. Impediments to Range Expansion

Numerous recent investigations have suggested that climate change, especially rapidly increasing global temperatures, has contributed to the range expansion of many arthropod vectors, including ticks [7,8,9,10,11,12]. Robust evidence is presented in these representative examples that range expansion is occurring. However, several factors—host availability (especially for preferred hosts) and host specificity, habitat suitability, relative humidity tolerance, the extent and duration of freezing temperatures, and human impact (habitat modification)—may limit the likely rate and extent of range expansion for different tick vector species. These factors need as much attention in understanding tick range expansion as has been given to climate change.

## 3. Selected North American Ticks and Range Expansion

### 3.1. American Dog Tick, Dermacentor variabilis 

This species is the primary vector for *Rickettsia rickettsii*—the bacterium causing Rocky Mountain spotted fever in humans and domestic or companion animals. It also is reportedly capable of transmitting *Coxiella burnetii* and *Francisella tularensis* [13]. Consequently, expansion of its geographic range poses a significant public health problem in areas previously free of this vector species [14]. 

**Figure 1:**
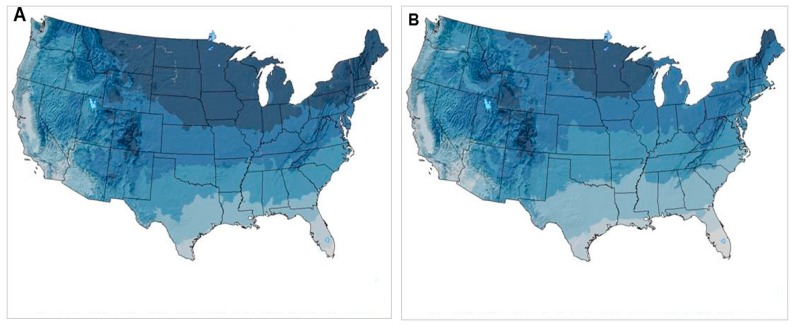
Maps showing the average minimum January temperatures (°F) in the continental United States: (**A**) 1970; (**B**) 2016. Dark Blue = –10 °F; medium blue = 11–20 °F; light blue = 21–30 °F; blue-green = 31–40 °F; gray = 41–50 °F. Photo credits Dr. R. Ryan Lash, Traveler’s Health Branch, DGMQ, Centers for Disease Control and Prevention, Atlanta, GA.

*Dermacentor variabilis* is a host-specific species for its juvenile stages which feed almost entirely on small mammals, but which also exhibits a broader host range for the adult stage, especially dogs and other medium-sized mammals [15]. *D. variabilis* is known as an “ambush” tick, i.e., ticks that cling to vegetation along woodland trails, roadsides and similar edge environments. This behavior limits their host range to those vertebrates that frequent these localities. Common hosts in the United States are meadow voles (*Microtus pennsylvanicus*) in grass and/or forb habitats and white-footed mice (*Peromyscus leucopus*) or deer mice (*P. maniculatus*) in the adjacent forests. This tick species has expanded its range northward into southern Canada, where it is reported established (and even abundant) in parts of Ontario, Saskatchewan, Manitoba [13] and from Nova Scotia [16]). As *D. variabilis* expands its range into more northern latitudes (as well as alpine terrains), it may not encounter these same rodents, essential for development of the larval and nymphal stage. Although meadow voles occur through most of the U.S. and Canada, the range of white-footed mice is more limited. In studies we did in Nova Scotia, red-backed voles, *Myodes gapperi*, served as substitute hosts in the woodland habitats in the absence of white-footed mice [16]. *M. gapperi* and its close relative *Myodes rutilis* are widespread throughout much of Canada, providing suitable hosts for larval and nymphal ticks in these regions [17]. In addition to host-dependent factors, *D. variabilis* is believed unable to survive over winter in regions where temperatures fall and remain well below the 0 °C mean winter isotherm (December through February), the period of sustained freezing conditions. However, a study of survival of unfed adults held under field conditions in Manitoba, Canada showed that an average of 20% of these ticks survived until April of the following year [18]. In studies by McEnroe [19], the 0 °C winter isotherm in that year was across southern Ohio and northern New Jersey to just north of Long Island, NY. However, climate change in the United States has resulted in milder winter temperatures since that time, with almost all the northeastern U.S. and much of the mid-western region averaging above freezing temperatures during the winter months (see Figure 1: maps for January temperatures for the year 2016 versus the year 1970). Thus, if current trends in climate change continue for the next several decades, and the average 10–20 °F January temperature expands northward, models predict that areas with suitable climate for *D. variabilis* could increase by approximately 50%, likely resulting in northward expansion throughout most of Canada [20]. However, it is not known whether suitable habitats, especially deciduous forests and hosts will also follow this trend.

### 3.2. Lone Star Tick, Amblyomma americanum 

*A. americanum* is a vector of *Ehrlichia chafeensis* and *E. ewingii* (the agents of human monocytic ehrlichiosis) and other potentially disease-causing agents such as *Rickettsia amblyommii* and *Borrelia lonestari.* Consequently, its increasing range expansion presents a serious public health threat in the northeastern United States and southern Canada [21]. In contrast to *D. variabilis*, *A. americanum* is regarded as a hunter tick, which will walk/crawl rapidly across many meters when attracted by host odors. This behavior enhances its host range since these ticks do not have to wait for a passing host. According to recent reports [11], lone star ticks have been expanding their zoogeographic range into new areas of the northern and mid-western United States. During the first half of the previous century, the northern limit of established populations of this species according those reports was approximately the Ohio river valley and extending northeast into southern New Jersey [11]. Subsequently, northward expansion has progressed to the extent that established populations have now been documented as far north as Michigan in the north central United States, as well as throughout Pennsylvania, almost all of New York state, and most counties of the New England states almost to the Canadian border. In the mid-western U.S., populations of lone star ticks have been reported as far west as Nebraska and South Dakota. According to those authors, the northward expansion of this tick’s geographic range is consistent with climate change. An interesting aspect of this rapid and very extensive range expansion concerns genetic changes in these remote populations. Using a novel genotyping by sequencing approach, Monzon et al. [11] showed that lone star ticks from New York and Oklahoma are genetically distinct from historic range populations in North and South Carolina. This also suggests the possibility of adaptive evolution in these distinct tick populations, with implications for their transmission of disease-causing agents. Lone star ticks are especially sensitive to changes in microclimatic conditions, primarily ground level moisture, which is strongly influenced by atmospheric relative humidity. Among the several climate predictor variables that influence the distribution of lone star ticks, July average atmospheric vapor pressure was the most important [21]. Arid and semi-arid habitats in the U.S. west of the 100th meridian will likely limit further westward range expansion while increasing global climate change causing increasingly dry summers may depress population densities in the southern parts of the range. Lone star ticks do not survive well in pine dominated habitats where they suffer increased saturation deficits during the late spring and summer months [22]. 

### 3.3. Gulf Coast Tick, Amblyomma maculatum

**Figure 2:**
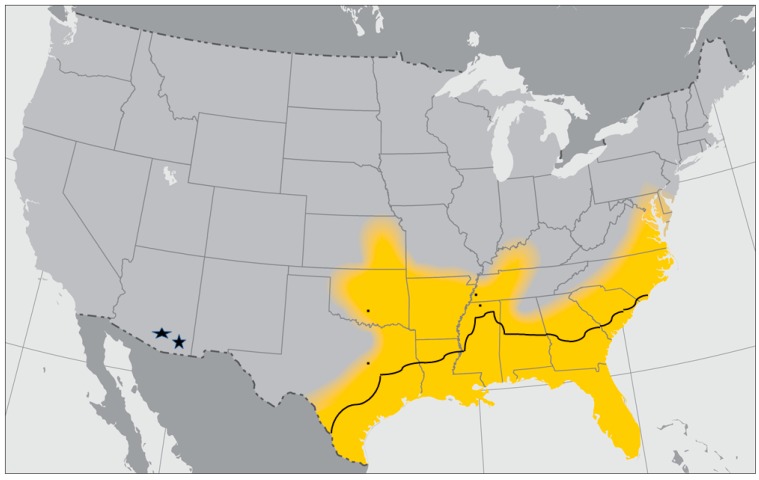
Map showing the current versus the historic geographic distribution of the Gulf Coast Tick, *Amblyomma maculatum* (yellow distribution based on [24] and CDC map). Dark black line and the four isolated black dots indicate the historic distribution based on Bishop and Trembley [32]. Asterisks in southeastern Arizona indicate new established populations as reported by Allerdice et al. [26]. Photo credit Dr. R. Ryan Lash, Traveler’s Health Branch, DGMQ, Centers for Disease Control and Prevention, Atlanta, GA.

The Gulf Coast tick is the vector of *Rickettsia parkeri*, a human pathogen capable of causing mild illness in infected people. Although *A. maculatum* serves as the primary vector for this rickettsial pathogen, spillover by co-feeding individuals into the more widespread *A. americanum* [23] greatly increases its importance as a public health threat. It is also the principal vector of *Hepatozoon americanum*, a pathogen infecting dogs [24]. *A*. *maculatum* is widely distributed in several Central and South American countries bordering the Gulf of Mexico and Caribbean Sea. In the United States, it was originally limited to the southeastern states bordering the Gulf of Mexico and the south Atlantic states. However, in the past several decades, its range has expanded northward into the mid-Atlantic states [24,25], in the Mid-west into Arkansas, Oklahoma, Kansas, and southwestern Tennessee [24] and, surprisingly, even as far west as southern Arizona [26] (Figure 2). The northward expansion is believed to be due to dispersal of immature ticks carried by migratory birds traveling to their arctic breeding grounds along the Atlantic flyway. Immatures of *A. maculatum* have been reported from more than 35 species of birds, mostly migratory species that regularly travel long distances [25,27]. During my research on ticks and tick-borne diseases in eastern Virginia during the period from 1963 to 2000, specimens of *A. maculatum* were quite rare and there was no evidence of breeding populations in the coastal islands. However, for the past two or three decades, these ticks are now well established throughout large areas of South and North Carolina, Virginia, eastern Maryland and Delaware. Its occurrence in the Appalachian regions in the western parts of these states is also believed to be related to migratory bird transport, since the Atlantic flyway is considered to include the Appalachian Mountains as well as the Atlantic coast. Its spread north into Oklahoma and Kansas was likely related to ticks attached to cattle relocated from the Gulf Coast states [24]. The ability of immature stages of this tick to feed on a wide variety of ground feeding birds, as well as various small mammals such as rats, mice and voles and the ability of the adult stages to feed on cattle, swine, white-tailed deer and other mammals suggest that finding suitable hosts will not be a factor limiting its northward and western range expansion. The spread north of feral pig populations and the continuing increase in white-tailed deer populations [24] are expected to contribute to further range expansion of this tick as well as intensification of its abundance in areas where it is already established.

It was expected that as warming trends continue, *A. maculatum* would expand northward along the Atlantic coast where it would encounter warm, moist microhabitats similar to its ancestral southern range since this species is intolerant of low relatively humidity. For Gulf Coast ticks, this would explain the recent establishment of breeding populations in coastal regions of the mid-Atlantic states. However, it does not explain breeding populations that have been found in northern Virginia, western North Carolina and even western Tennessee and Kentucky [24,28] where environmental conditions are cooler and not as humid. Similarly, it does not explain establishment of breeding populations in the more arid pasture environments of Oklahoma, Kansas, and xeric environments of southern Arizona, for the same reasons noted above. It is not known whether these findings indicate that the newly dispersed populations have evolved greater tolerance to the cooler and drier local environments, or whether they are surviving in isolated pockets in warmer, more humid habitats along local rivers and creeks. 

Remarkably, several studies have reported little genetic variability and high levels of gene flow among expanding populations of *A. maculatum* [29]. Ferrari et al. [30] suggested expansion of this tick along a migrating front rather than as long-standing sporadic, isolated populations. Nadolny et al. [31] used genetic analysis to track the ancestry of newly established populations. Their findings showed diverse haplotypes but there was no significant relationship to geographic distribution and little or no apparent connectivity between the many different localities. Especially important is that the genetic population structures were unique, i.e., there was little gene flow between sampling localities, which these authors suggest indicates that the localized tick populations resulted from separate founder events. 

### 3.4. Black-Legged Tick, Ixodes scapularis

This species is the primary vector for a greater variety of tick-borne diseases than any human or animal biting tick in North America, including the bacteria causing Lyme disease, human granulocytic anaplasmosis, tick-borne relapsing fever, human babesiosis, ehrlichiosis [1] and the virus causing Powassan illness. Since it was first recognized as the vector of *Borrelia burgdorferi* (sensu strictu), the etiological agent of Lyme disease, in the 1970s, the geographic range of this tick has expanded substantially [8,9,33], likely mostly due to the moderating winter temperatures that have occurred over the past few decades (Figure 1). Although previously present in modest numbers and scattered populations by the end of the 19th century, *I. scapularis* has largely spread throughout most of the eastern United States and into southern Canada during the 20th century following the reforestation and reintroduction and proliferation of white-tailed deer [5]. Tick population densities have also intensified in many of the recolonized localities. The number of counties in which it is believed to be established has increased greatly throughout almost all of the United States east of the Mississippi river as well as most of the midwestern U.S., up to approximately the 95° W longitude [34]. It is also established in parts of southern Canada and appears to be advancing rapidly, e.g., at ~46 km/year in Ontario, although colonization of different habitats is slower and heterogeneous (7). *I. scapularis* has also spread into Canada’s Quebec province, where the numbers of ticks collected increased more than four-fold over the six-year period from 2008 to 2014, along with an increasing geographic range [35]. Remarkably, given the sensitivity of *I. scapularis* to desiccation, a recent report shows evidence of westward expansion into prairie grasslands in Iowa [36] and parts of the southeastern Canadian Prairie region [37].

These generalizations about range expansion are important but do not tell the whole story. Many earlier studies of *I. scapularis* distribution were based on field reports at the county level, defining the tick’s spatial distribution on political maps [38]. However, availability of suitable habitats is also important. *I. scapularis* is adapted to forest and brushy habitats, especially deciduous forests where sufficient ground cover provides very humid microenvironments. *I. scapularis* is extremely sensitive to desiccation, a factor limiting the duration of its above ground questing activity. Localities with suitable habitat largely determine whether this tick can expand its range. In Minnesota, for example, the majority of the more suitable habitats for *I. scapularis* establishment are areas dominated by cool temperate forests, areas representing 19% of the state [39]. *I. scapularis* may become established in other less suitable habitats, e.g., temperate shrublands. More to the point, these studies also suggest that the cold boreal forests, especially the Laurentian-Acadian and spruce-fir forests characteristic of the northern-most region of Minnesota, Wisconsin and adjacent areas of southern Canada are unlikely to support *I. scapularis* range expansion. 

Cold hardiness is an important factor affecting range expansion. Data on this subject is limited, but the few studies available suggest that *I. scapularis* can survive brief episodes of temperatures as low as –10 °C. for roughly two hours. This low temperature tolerance is well below measurements of subzero temperatures in leaf litter and underlying soil surfaces [40]. Cold hardiness in these ticks is also enhanced by the presence of so-called antifreeze proteins, namely *I. scapularis* antifreeze glycoproteins (AFGP). These proteins are typically large glycoproteins that bind ice crystals. Remarkably, infection with *Anaplasma phagocytophilum* enhances expression of IAFGP, so that *A. phagocytophilum*-infected ticks are more likely to survive freezing episodes than uninfected ticks [41]. 

Host range is not likely to be a major limiting factor in the range expansion of *I. scapularis*, since this is a highly permissive species. The most common hosts for the adults are white-tailed deer (*Odocoileus virginianus*), but numerous other large and medium sized mammals, including humans are also attacked; for the immature stages, the most common hosts are small mammals, especially white-footed mice (*Peromyscus leucopus*) and deer mice (*Peromyscus maniculatus*). However, *I. scapularis* larvae and nymphs also feed on other small mammals, as well as ground feeding birds and even lizards [5]. Consequently, these ticks are likely to find suitable hosts as their geographic range expands. However, bird migration also plays a key role in this process. According to [42], migratory birds flying north during the spring migration disperse 50–175 million *I. scapularis* ticks across wide areas of Canada. During the spring migration period, immature *I. scapularis* dropped from these birds contributed to establishing new breeding populations in sites where the local habitats were suboptimal for their survival in the absence of migrating birds. They also contributed to boosting tick population density in already established populations [43].

Also important is the increasing abundance of *I. scapularis* populations within localities where they are already established. For example, in Rhode Island, collections of nymphal *I. scapularis* (nymphs/h) in 1994 were rated as medium to high only in the southwestern part of the state, west of Narraganset Bay and were low or absent in the north. Areas with medium to high population densities have spread north and east since that time; by 2014, the last year for which data are available, they now cover the entire state. Maps illustrating these changes in increasing tick population densities can be found at the Tick Encounter website (http://www.tickencounter.org).

## 4. Conclusions

This review summarizes evidence demonstrating that the geographic ranges of all four ixodid tick species discussed in this review are expanding. In addition to their expansion northward, they are also moving westward into drier areas of the North American continent previously considered inhospitable for their establishment. Perhaps the most surprising example of tick adaptability to habitats in these semi-arid regions is the Gulf Coast tick, once limited almost exclusively to the Gulf Coast and south Atlantic states, that has expanded northward along the U.S. Atlantic coast, and also as far west as Arizona. The *A. maculatum* populations in southern Arizona illustrate another phenomenon, namely, that what is currently perceived as range expansion may in fact represent relic populations that have persisted in highly specific biomes [26]. 

In addition to suitable climate and habitats, tick range expansion is host dependent. Even small numbers of ticks may be sufficient to establish a new population so long as competent hosts and suitable habitats and temperatures are present [42,43].

Predictions about tick range expansion also assume the stability of the major biomes, each comprising the same life-form. However, this is uncertain, especially since human activities have greatly increased the amount of land areas used for crop production and grazing of domestic herbivores. Human-led disturbance of the natural environment is likely to continue, leading to increasing range expansion of grasslands and croplands at the expanse of forests and shrublands [44].
